# [μ-1,2-Bis(diphenyl­phosphan­yl)-1,2-dimethyl­hydrazine-κ^2^ *P*:*P*′]bis­[chlorido­gold(I)]

**DOI:** 10.1107/S1600536810050506

**Published:** 2010-12-08

**Authors:** Frederik H. Kriel, Manuel A. Fernandes, Judy Coates

**Affiliations:** a Project AuTEK, Mintek, Private Bag X3015, Randburg 2125, South Africa; b Molecular Science Institute, School of Chemistry, University of the Witwatersrand, PO Wits, 2050 Johannesburg, South Africa

## Abstract

The title compound, [Au_2_Cl_2_(C_26_H_26_N_2_P_2_)], is formed from a bidentate phosphine ligand complexed to two linearly coordinated gold(I) atoms. The gold(I) atoms are 3.4873 (7) Å apart. The mol­ecule exhibits a crystallographic twofold rotation axis.

## Related literature

For the structure of the parent ligand, see: Kriel *et al.* (2010*a* ▶). For the synthesis of the parent ligand and related structures utilising alternative metals, see: Reddy *et al.* (1994 ▶, 1995 ▶); Kriel *et al.* (2010*b* ▶). For Au⋯Au inter­actions, see: Holleman & Wiberg (2001 ▶). For related gold structures of dppe and dppen (dppe = 1,2-bis­(diphenyl­phosphino)ethane; dppen = 1,2-bis­(diphenyl­phosphino)ethene), see: Eggleston *et al.* (1985 ▶) and Jones (1980 ▶), respectively.
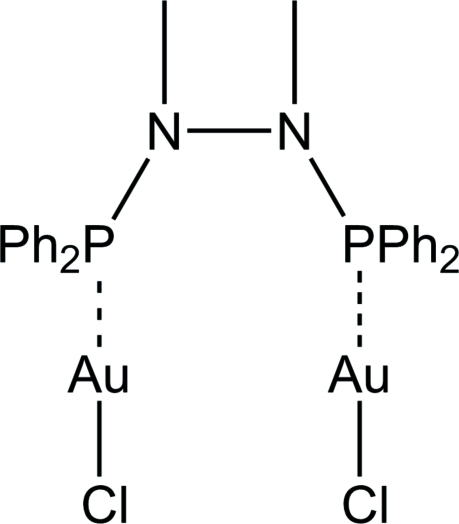


## Experimental

### 

#### Crystal data

[Au_2_Cl_2_(C_26_H_26_N_2_P_2_)]
*M* *_r_* = 893.26
Tetragonal, 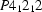

*a* = 10.6720 (14) Å
*c* = 23.439 (4) Å
*V* = 2669.5 (7) Å^3^
*Z* = 4
Mo *K*α radiation
μ = 11.32 mm^−1^
*T* = 173 K
0.18 × 0.10 × 0.08 mm

#### Data collection

Bruker SMART CCD area-detector diffractometer
Absorption correction: integration (*SADABS*; Bruker, 1999 ▶) *T*_min_ = 0.294, *T*_max_ = 0.457
25347 measured reflections
3312 independent reflections
2709 reflections with *I* > 2σ(*I*)
*R*_int_ = 0.091

#### Refinement

*R*[*F* ^2^ > 2σ(*F* ^2^)] = 0.030
*wR*(*F* ^2^) = 0.056
*S* = 0.98
3312 reflections
154 parameters
H-atom parameters constrained
Δρ_max_ = 1.43 e Å^−3^
Δρ_min_ = −0.78 e Å^−3^
Absolute structure: Flack (1983 ▶), 1332 Friedel pairs
Flack parameter: 0.011 (10)

### 

Data collection: *SMART-NT* (Bruker, 1998 ▶); cell refinement: *SAINT-Plus* (Bruker, 1999 ▶); data reduction: *SAINT-Plus*; program(s) used to solve structure: *SHELXS97* (Sheldrick, 2008 ▶); program(s) used to refine structure: *SHELXL97* (Sheldrick, 2008 ▶); molecular graphics: *ORTEP-3* (Farrugia, 1997 ▶) and *Mercury* (Macrae *et al.*, 2008) ▶; software used to prepare material for publication: *WinGX* (Farrugia, 1999 ▶).

## Supplementary Material

Crystal structure: contains datablocks I, global. DOI: 10.1107/S1600536810050506/br2152sup1.cif

Structure factors: contains datablocks I. DOI: 10.1107/S1600536810050506/br2152Isup2.hkl

Additional supplementary materials:  crystallographic information; 3D view; checkCIF report

## Figures and Tables

**Fig. 1.**
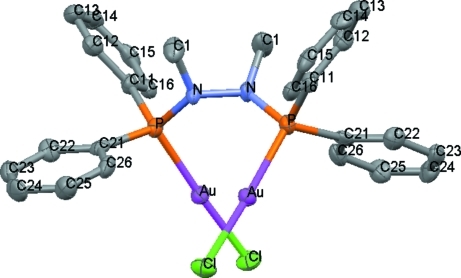
Molecular structure of the title compound drawn with displacement ellipsoids at the 50% probability level. Hydrogen atoms have been omitted for clarity.

**Fig. 2.**
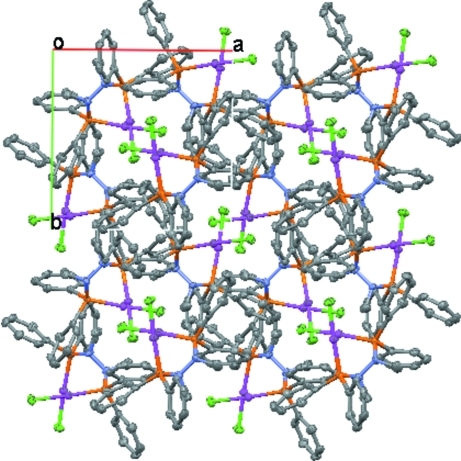
Packing of the title compound as seen down the c axis.

## supplementary crystallographic information

### Comment

In the title compound, gold(I) forms an almost linear complex with a P—Au—Cl angles of 176.34 °. The Au—Au distances is 3.487 Å and is slightly too long to be classified as an aurophilic interaction, which is defined as between 2.7 Å and 3.4 Å (Holleman *et al.*, 2001). Other bond lengths are within expected ranges.

A direct comparison of the title compound and the analogous dppe complex (where dppe = 1,2-bis(diphenylphosphino)ethane); ClAu(dppe)AuCl shows that the preference for the *gauche* conformation of the hydrazine backbone in the parent ligand (Kriel *et al.*) may explain the observed intramolecular Au—Au interactions as compared to the intermolecular Au—Au interactions observed in the two polymorphs of ClAu(dppe)AuCl (3.189 Å and 3.221 Å ). The formation of intermolecular Au—Au interactions between dimers of ClAu(dppe)AuCl may be attributed to the different conformation of the ethyl backbone as illustrated by the torsion angles of the two polymorphs (-18.6 ° and 50.7 °) (Eggleston *et al.*, 1985). Intermolecular Au—Au contacts are also observed for the analogous ClAu(dppen)AuCl complex (dppen = 1,2–bis(diphenylphosphino)ethene) (3.05 Å), where the ethene bridge is constrained to a *cis* conformation by the double bond (Jones, 1980).

The insolubility of the title compound in both non polar and highly polar solvents once crystallized may be a result of the tightly packed parallel helixes, that are formed in the solid state. While the complex readily crystallizes from THF it does not include THF in the structure. This structure exhibits a α-helical packing down the *c* axis (Figure 2). This unique packing results in parallel helixes that have no voids large enough to include solvent and leads to a stabilizing short contact distance of 2.900 Å between Cl and H(15).

### Experimental

General procedure

Tetrahydrothiophenegold(I) chloride [(THT)AuCl] was suspended in tetrahydrofuran. 0.5 equivalents of the ligand, bis(diphenylphosphino)-1,2-dimethylhydrazine, dissolved in dicloromethane was added to the stirred suspension. The suspension turned yellow and after a short time micro crystals started to form. The solvent was removed *in vacuo* to afford the product as a micro-crystalline powder.

Alternatively, the reaction was carried out in dichloromethane to afforded the title compound. By addition of a few drops of tehrahydrofuran it was possible to grow crystals overnight.

### Refinement

The H atoms were positioned geometrically and allowed to ride on their respective parent atoms, with C—H = 0.93 (Ar—H) or 0.96 (CH_3_) Å, and with *U*_eq_ = 1.2 (Ar—H) or 1.5 (CH_3_) *U*_eq_(C).

### Crystal data

**Table:**

[Au_2_Cl_2_(C_26_H_26_N_2_P_2_)]	*D*_x_ = 2.223 Mg m^−^^3^
*M**_r_* = 893.26	Mo *K*α radiation, λ = 0.71073 Å
Tetragonal, *P*4_1_2_1_2	Cell parameters from 7602 reflections
Hall symbol: P 4abw 2nw	θ = 5.2–49.5°
*a* = 10.6720 (14) Å	µ = 11.32 mm^−^^1^
*c* = 23.439 (4) Å	*T* = 173 K
*V* = 2669.5 (7) Å^3^	Prismic, colourless
*Z* = 4	0.18 × 0.10 × 0.08 mm
*F*(000) = 1672	

### Data collection

**Table:**

Bruker SMART CCD area-detector diffractometer	3312 independent reflections
Radiation source: fine-focus sealed tube	2709 reflections with *I* > 2σ(*I*)
graphite	*R*_int_ = 0.091
phi and ω scans	θ_max_ = 28.3°, θ_min_ = 2.1°
Absorption correction: integration (*SADABS*; Bruker, 1999)	*h* = −14→14
*T*_min_ = 0.294, *T*_max_ = 0.457	*k* = −11→14
25347 measured reflections	*l* = −31→31

### Refinement

**Table:**

Refinement on *F*^2^	Secondary atom site location: difference Fourier map
Least-squares matrix: full	Hydrogen site location: inferred from neighbouring sites
*R*[*F*^2^ > 2σ(*F*^2^)] = 0.030	H-atom parameters constrained
*wR*(*F*^2^) = 0.056	*w* = 1/[σ^2^(*F*_o_^2^) + (0.0218*P*)^2^] where *P* = (*F*_o_^2^ + 2*F*_c_^2^)/3
*S* = 0.98	(Δ/σ)_max_ < 0.001
3312 reflections	Δρ_max_ = 1.43 e Å^−^^3^
154 parameters	Δρ_min_ = −0.78 e Å^−^^3^
0 restraints	Absolute structure: Flack (1983), 1332 Friedel pairs
Primary atom site location: structure-invariant direct methods	Flack parameter: 0.011 (10)

### Special details

**Table:**

Experimental. Reaction: bis(diphenylphosphino)-1,2-dimethylhydrazine: 146 mg (0.34 mmol), (THT)AuCl: 200 mg (0.68 mmol), THF: 2 ml, DCM: 5 ml, Yield: 89% Grey crystals or white precipitate. Crystals are insoluble in organic and highly polar solvents. ^1^H NMR: (CDCl_3_, 300 MHz) δH 7.85 (dd, Arom, J (^1^H-^31^P) = 13.2, J (^1^H-^1^H) = 8.1), 7.52 (t, Arom, J (^1^H-^1^H) = 9.40 Hz), 7.40 (dd, Arom, J (^1^H-^31^P) = 17.7, J (^1^H-^1^H) = 7.4), 2.76 (d, CH3, ^3^J = 7.8 Hz). ^13^C NMR: Compound too insoluble in NMR solvents. ^31^P NMR: (CDCl_3_, 121 MHz) δP 87.1. MS: No useful information could be obtained. EA: Calc: (Au_2_Cl_2_P_2_N_2_C_26_H_26_) C 34.96%, H 2.93%, N 3.14% Found: C 35.29%, H 2.93%, N 3.13%. MP: 228 - 230 °C.
Geometry. All esds (except the esd in the dihedral angle between two l.s. planes) are estimated using the full covariance matrix. The cell esds are taken into account individually in the estimation of esds in distances, angles and torsion angles; correlations between esds in cell parameters are only used when they are defined by crystal symmetry. An approximate (isotropic) treatment of cell esds is used for estimating esds involving l.s. planes.
Refinement. Refinement of F^2^ against ALL reflections. The weighted R-factor wR and goodness of fit S are based on F^2^, conventional R-factors R are based on F, with F set to zero for negative F^2^. The threshold expression of F^2^ > 2sigma(F^2^) is used only for calculating R-factors(gt) etc. and is not relevant to the choice of reflections for refinement. R-factors based on F^2^ are statistically about twice as large as those based on F, and R- factors based on ALL data will be even larger.

### Fractional atomic coordinates and isotropic or equivalent isotropic displacement parameters (Å^2^)

**Table:**

	*x*	*y*	*z*	*U*_iso_*/*U*_eq_	
C1	0.0733 (7)	0.2483 (7)	−0.0265 (3)	0.0412 (18)	
H1A	0.0168	0.3183	−0.0269	0.062*	
H1B	0.0377	0.1816	−0.0043	0.062*	
H1C	0.0871	0.2199	−0.0649	0.062*	
C11	0.1070 (6)	0.3372 (6)	0.1127 (2)	0.0242 (14)	
C12	−0.0234 (7)	0.3312 (6)	0.1112 (2)	0.0272 (16)	
H12	−0.0658	0.3546	0.0782	0.033*	
C13	−0.0888 (7)	0.2919 (6)	0.1571 (3)	0.0367 (17)	
H13	−0.1758	0.2884	0.1555	0.044*	
C14	−0.0262 (7)	0.2563 (6)	0.2072 (3)	0.0378 (19)	
H14	−0.0719	0.2331	0.2393	0.045*	
C15	0.1029 (8)	0.2556 (6)	0.2089 (2)	0.0325 (17)	
H15	0.1450	0.2271	0.2411	0.039*	
C16	0.1693 (6)	0.2981 (6)	0.1618 (3)	0.0277 (16)	
H16	0.2564	0.3006	0.1630	0.033*	
C21	0.1195 (6)	0.5279 (6)	0.0255 (3)	0.0248 (15)	
C22	0.0479 (6)	0.6049 (6)	0.0614 (2)	0.0315 (16)	
H22	0.0321	0.5804	0.0988	0.038*	
C23	0.0009 (7)	0.7174 (7)	0.0411 (3)	0.0358 (18)	
H23	−0.0455	0.7687	0.0652	0.043*	
C24	0.0220 (7)	0.7542 (7)	−0.0142 (3)	0.0387 (19)	
H24	−0.0114	0.8291	−0.0276	0.046*	
C25	0.0927 (7)	0.6797 (6)	−0.0496 (3)	0.0356 (17)	
H25	0.1082	0.7052	−0.0868	0.043*	
C26	0.1405 (6)	0.5680 (7)	−0.0303 (3)	0.0311 (16)	
H26	0.1877	0.5184	−0.0549	0.037*	
N	0.1921 (5)	0.2864 (5)	−0.0014 (2)	0.0235 (12)	
P	0.19968 (15)	0.39132 (16)	0.05313 (6)	0.0224 (4)	
Cl	0.60720 (16)	0.45361 (16)	0.09865 (6)	0.0346 (4)	
Au	0.40052 (2)	0.42768 (2)	0.073875 (9)	0.02472 (7)	

### Atomic displacement parameters (Å^2^)

**Table:**

	*U*^11^	*U*^22^	*U*^33^	*U*^12^	*U*^13^	*U*^23^
C1	0.033 (5)	0.046 (5)	0.044 (4)	0.007 (4)	−0.011 (4)	−0.019 (4)
C11	0.024 (4)	0.023 (3)	0.025 (3)	0.005 (3)	0.001 (3)	0.004 (3)
C12	0.033 (4)	0.026 (4)	0.023 (4)	0.001 (3)	−0.002 (3)	0.004 (3)
C13	0.028 (4)	0.028 (4)	0.054 (4)	−0.002 (4)	0.004 (4)	0.003 (3)
C14	0.043 (5)	0.026 (4)	0.045 (4)	−0.010 (4)	0.016 (4)	0.003 (3)
C15	0.057 (5)	0.019 (3)	0.021 (3)	0.009 (4)	−0.003 (3)	0.003 (3)
C16	0.027 (4)	0.022 (4)	0.035 (4)	0.002 (3)	0.002 (3)	−0.008 (3)
C21	0.015 (3)	0.028 (4)	0.031 (3)	−0.005 (3)	−0.008 (3)	0.003 (3)
C22	0.036 (4)	0.034 (4)	0.025 (3)	0.003 (4)	−0.007 (3)	−0.003 (3)
C23	0.027 (4)	0.032 (4)	0.048 (5)	0.006 (3)	−0.001 (3)	−0.015 (4)
C24	0.039 (5)	0.022 (4)	0.055 (5)	0.006 (4)	−0.009 (4)	0.009 (4)
C25	0.034 (4)	0.037 (4)	0.036 (4)	0.004 (4)	−0.001 (3)	0.014 (3)
C26	0.021 (3)	0.040 (4)	0.032 (4)	0.006 (3)	0.002 (3)	−0.007 (4)
N	0.019 (3)	0.024 (3)	0.027 (3)	0.007 (2)	−0.002 (2)	−0.006 (2)
P	0.0200 (8)	0.0247 (10)	0.0226 (7)	0.0048 (8)	−0.0003 (6)	−0.0011 (7)
Cl	0.0263 (9)	0.0423 (10)	0.0353 (8)	−0.0062 (8)	−0.0089 (7)	0.0025 (7)
Au	0.02271 (14)	0.02537 (15)	0.02609 (10)	0.00065 (11)	−0.00336 (11)	−0.00038 (11)

### Geometric parameters (Å, °)

**Table:**

C1—N	1.455 (8)	C21—C26	1.394 (8)
C1—H1A	0.9600	C21—C22	1.402 (9)
C1—H1B	0.9600	C21—P	1.811 (6)
C1—H1C	0.9600	C22—C23	1.386 (9)
C11—C16	1.393 (8)	C22—H22	0.9300
C11—C12	1.394 (9)	C23—C24	1.372 (9)
C11—P	1.805 (6)	C23—H23	0.9300
C12—C13	1.349 (9)	C24—C25	1.374 (9)
C12—H12	0.9300	C24—H24	0.9300
C13—C14	1.404 (9)	C25—C26	1.373 (9)
C13—H13	0.9300	C25—H25	0.9300
C14—C15	1.379 (10)	C26—H26	0.9300
C14—H14	0.9300	N—N^i^	1.425 (10)
C15—C16	1.388 (8)	N—P	1.702 (5)
C15—H15	0.9300	P—Au	2.2318 (16)
C16—H16	0.9300	Cl—Au	2.2976 (17)
			
N—C1—H1A	109.5	C22—C21—P	120.9 (5)
N—C1—H1B	109.5	C23—C22—C21	119.9 (6)
H1A—C1—H1B	109.5	C23—C22—H22	120.1
N—C1—H1C	109.5	C21—C22—H22	120.1
H1A—C1—H1C	109.5	C24—C23—C22	120.8 (6)
H1B—C1—H1C	109.5	C24—C23—H23	119.6
C16—C11—C12	118.9 (6)	C22—C23—H23	119.6
C16—C11—P	118.2 (5)	C23—C24—C25	119.6 (6)
C12—C11—P	122.9 (5)	C23—C24—H24	120.2
C13—C12—C11	120.8 (6)	C25—C24—H24	120.2
C13—C12—H12	119.6	C26—C25—C24	120.5 (6)
C11—C12—H12	119.6	C26—C25—H25	119.7
C12—C13—C14	120.3 (7)	C24—C25—H25	119.7
C12—C13—H13	119.8	C25—C26—C21	121.0 (6)
C14—C13—H13	119.8	C25—C26—H26	119.5
C15—C14—C13	120.0 (6)	C21—C26—H26	119.5
C15—C14—H14	120.0	N^i^—N—C1	115.9 (4)
C13—C14—H14	120.0	N^i^—N—P	113.3 (5)
C14—C15—C16	119.1 (6)	C1—N—P	121.9 (4)
C14—C15—H15	120.4	N—P—C11	110.1 (3)
C16—C15—H15	120.4	N—P—C21	103.8 (3)
C15—C16—C11	120.8 (6)	C11—P—C21	106.0 (3)
C15—C16—H16	119.6	N—P—Au	108.89 (18)
C11—C16—H16	119.6	C11—P—Au	114.4 (2)
C26—C21—C22	118.1 (6)	C21—P—Au	113.1 (2)
C26—C21—P	120.4 (5)	P—Au—Cl	176.34 (6)
			
C16—C11—C12—C13	2.0 (10)	C1—N—P—C11	56.8 (6)
P—C11—C12—C13	−179.0 (5)	N^i^—N—P—C21	157.7 (3)
C11—C12—C13—C14	0.0 (10)	C1—N—P—C21	−56.3 (6)
C12—C13—C14—C15	−3.1 (10)	N^i^—N—P—Au	37.0 (4)
C13—C14—C15—C16	4.1 (10)	C1—N—P—Au	−177.0 (5)
C14—C15—C16—C11	−2.1 (9)	C16—C11—P—N	110.1 (5)
C12—C11—C16—C15	−0.9 (9)	C12—C11—P—N	−69.0 (6)
P—C11—C16—C15	180.0 (5)	C16—C11—P—C21	−138.3 (5)
C26—C21—C22—C23	−0.2 (10)	C12—C11—P—C21	42.7 (6)
P—C21—C22—C23	171.6 (5)	C16—C11—P—Au	−13.0 (6)
C21—C22—C23—C24	0.9 (10)	C12—C11—P—Au	168.0 (5)
C22—C23—C24—C25	−1.3 (11)	C26—C21—P—N	−42.6 (6)
C23—C24—C25—C26	1.1 (11)	C22—C21—P—N	145.7 (5)
C24—C25—C26—C21	−0.5 (10)	C26—C21—P—C11	−158.7 (5)
C22—C21—C26—C25	0.0 (9)	C22—C21—P—C11	29.7 (6)
P—C21—C26—C25	−171.8 (5)	C26—C21—P—Au	75.2 (5)
N^i^—N—P—C11	−89.2 (4)	C22—C21—P—Au	−96.5 (5)

Symmetry codes: (i) *y*, *x*, −*z*.
